# Ensuring effective transitions in care for hospitalized people living with HIV

**DOI:** 10.3389/fpubh.2026.1808136

**Published:** 2026-04-30

**Authors:** Ajay Rangaraj, Nathan Ford, Alena Kamenshchikova

**Affiliations:** 1 Department of HIV, Tuberculosis, Hepatitis, and STIs, World Health Organization, Geneva, Switzerland; 2 Centre for Integrated Data and Epidemiological Research, University of Cape Town, Cape Town, South Africa; 3 Faculty of Health, Medicine, and Life Sciences, Maastricht University, Maastricht, Netherlands

**Keywords:** discharge, HIV, morbidity, mortality, readmission

## Abstract

Recent changes in the global health financing resulted in a steep reduction in donor support for HIV programmes worldwide, threatening years of progress in reducing infections and mortality. These developments require existing HIV programmes to rethink their priorities. Advanced HIV disease (AHD) is a condition present in approximately 30% of people living with HIV (PLHIV) presenting to HIV care programmes worldwide. These individuals are defined by their low immune status, having a CD4 cell count ≤200 cells/mm^3^ or World Health Organization (WHO) Clinical Stage 3 or 4 AIDS-defining illness—and are at significantly greater risk of hospitalization and poor outcomes following discharge from hospital. A recent systematic review found that in low-and middle-income settings, 19% of those discharged from hospital were subsequently readmitted, and 14% died. Post-discharge outcomes are an important and often poorly acknowledged gap in the HIV care cascade where PLHIV fail to successfully return to regular care services. In this mini-review, we provide an overview on post-discharge outcomes for PLHIV and suggest a way forward for expanding current service delivery approaches to improve outcomes. The focus is on low-income settings, but reflects broader trends on post-discharge outcomes for PLHIV.

## 1 Introduction

Recent changes in the global health financing resulted in a steep reduction in donor support for HIV programmes worldwide, threatening years of progress in reducing infections and mortality. These developments require existing HIV programmes to rethink their priorities. Advanced HIV disease (AHD) is a condition present in approximately 30% of people living with HIV (PLHIV) presenting to HIV care programmes worldwide (1). These individuals are defined by their low immune status, having a CD_4_ cell count ≤200 cells/mm^3^ or World Health Organization (WHO) Clinical Stage 3 or 4 AIDS-defining illness (2)—and are at significantly greater risk of hospitalization and poor outcomes following discharge from hospital. A recent systematic review found that in low- and middle- income settings, 19% of those discharged from hospital were subsequently readmitted, and 14% died (3). Post-discharge outcomes are an important and often poorly acknowledged gap in the HIV care cascade where PLHIV fail to successfully return to regular care services.

In this mini-review, we provide an overview on post-discharge outcomes for PLHIV and suggest a way forward for expanding current service delivery approaches to improve outcomes. The focus is on low-income settings, but reflects broader trends on post-discharge outcomes for PLHIV.

## 2 The link between AHD, inpatient admissions and poor post-discharge outcomes

Globally, over 40% of all admissions among PLHIV are due to AIDS-related causes, with little change since 2014 (4). Tuberculosis (TB), bacterial infections and fungal infections are major contributors to these AIDS-related causes (4). Indirect evidence from published literature indicate that HIV has a strong association with cases of sepsis and severe sepsis, and is a primary driver of inpatient mortality in high burden, resource-limited settings (5–7). Management of AHD is often challenging in low- and middle-income settings—workforce limitations and limited access to appropriate diagnostics and therapeutics for treating opportunistic infections, are some examples of the challenges faced in these settings (8, 9). The predominance of TB as a cause of admission among PLHIV is important, as the presence of disseminated TB at admission is a strong predictor of 30-day mortality (10). A diagnosis of extra-pulmonary TB (e.g., TB meningitis) or cryptococcal meningitis does not allow for immediate initiation of antiretroviral therapy (ART) due to high risk of immune reconstitution inflammatory syndrome, which can be life-threatening. ART is a life-saving measure but is withheld for 4–6 weeks, after which ART can be initiated (11). An individual diagnosed with cryptococcal meningitis will begin treatment as an inpatient, continue treatment upon returning home for several weeks, then need to be linked back to care in order to start ART. Such cycling in and out of the health system can result in an increased risk of loss to care and death.

The poor post-discharge outcomes reported across diverse settings, including those with limited resources (3) raise concerns about the care continuum. Re-engaging PLHIV in care services following hospital discharge is not straightforward, particularly if HIV diagnosis occurs during their inpatient stay. Hospital admissions with unknown HIV status result in worse outcomes, as they are more likely to have poor immune status (12, 13). PLHIV face a high risk of mortality even while in hospital (approximately one in six PLHIV dies in hospital), and there has been limited research to identify interventions that reduce death among PLHIV admitted to hospital (14). Sepsis is also common among PLHIV in intensive care units (ICU) (6, 7, 15), where individuals frequently require supportive services both during inpatient stay and following discharge. A range of important short- and long-term health consequences arise with sepsis, such as post-ICU syndrome and/or post-sepsis syndrome, diminished functional and cognitive status, immune and metabolic dysfunction, cardiovascular complications and kidney injury (16, 17). Bloodstream infections (including HIV and malaria) are the leading infectious syndromes complicating sepsis-related deaths from non-infectious causes (15).

However, HIV programmes primarily serve outpatient populations and often lack the capacity to track transitions to and from inpatient care—data systems in most low resource, high-HIV burden countries face challenges of consolidating data across the health system, with data inconsistency a prominent challenge (18, 19). A recent survey of 21 African countries found that only 4 countries were able to demonstrate capacity to provide comprehensive care, laboratory and monitoring services for AHD. The survey also reported that uptake of AHD policies was slow in the majority of countries, as of 2024 (20).

Thus, the persistence of AHD and AIDS-related admissions highlight gaps in service provision, linkage to care and data collection and reporting across the health system.

## 3 How can expanding service delivery approaches help?

Models of effective HIV care have evolved substantially in the last decade. Differentiated service delivery has emerged as a key programmatic approach to help tailor care to populations with different needs. WHO recommendations address a range of services, including when and where to start ART, how frequently to provide medicine refills, support for adherence and re-engagement, and the integration of non-communicable disease and mental health services. This differentiated service delivery approach was primarily developed for outpatient settings, but should consider inpatient care (21–23). In 2025, WHO introduced new recommendations to support PLHIV transitioning to outpatient care and reduce avoidable readmissions. The recommendation was informed by a systematic review of interventions for improving outcomes at discharge (11), and includes pre-discharge goal setting, medication review, transitional care planning, telephone follow-up, home visits by healthcare providers or peer support groups (or in combination) and individualized support. These interventions were found to reduce unnecessary readmissions and improve linkage/retention in care, but did not reduce mortality; some PLHIV in the included studies were reported to have died before receiving study interventions (11, 24). More research is urgently needed in order to identify interventions that work to reduce mortality both in hospital and after discharge.

To ensure successful care transitions, planning for discharge and pre-discharge goal setting should begin as soon as recovery begins while in hospital. Some important examples of interventions in the pre-discharge period include case referrals ahead of discharge; initiation of peer supporter sessions; risk stratification for readmission; medication review; provision of a discharge card with clear instructions for laboratory tests, medications, and follow-up instructions; goal setting with a nurse along with a review of barriers to care; patient education and development of a transitional care plan, ideally in the context of a multidisciplinary team (25–33).

The post-discharge period is a highly varied and challenging period, particularly for those recovering from an ICU stay (34). Once PLHIV return home, counselling from healthcare workers and patient care navigators is important to ensure adherence to their prescription at discharge. Additionally, establishing appointment schedules and escorting PLHIV with impaired functional status to clinic visits are standard supportive actions. These activities can be further strengthened with provision of telephone follow-up calls (25, 27, 29, 30, 32), sharing information brochures (26, 32), follow-up home visits (25–27, 35), motivational interviews (26), macronutrient supplements for those who are food insecure (27), appointment reminders, assisted transport with patient care navigators as well as medication reconciliation by pharmacist at the discharge clinic.

Home visits also present an important opportunity for healthcare workers to alleviate health-related suffering, using a palliative care approach to help improve quality of life and facilitate further recovery. This includes sensitization of healthcare workers towards symptom recognition, pain assessment and measures to help alleviate pain in those recovering from serious illness. These visits can facilitate linkages with spiritual counsellors where relevant in a given setting, thus providing a more holistic approach to post-discharge care (36).

PLHIV discharged from hospital may not attend previously scheduled clinic visits for several weeks, and may be recorded as having missed a clinic visit or potentially be lost to care. It is at this stage where PLHIV who were previously engaged in care services but subsequently fell ill need to be traced and re-engaged in services. This also presents an opportunity to initiate or reinitiate ART outside of health facilities and provide adherence support interventions. This integrative approach can help overcome additional barriers that PLHIV may face, and the reasons for care disengagement are likely similar to those who disengage from care as outpatients—psychological, mental health challenges; lack of family or social support; mobility issues; acute proximal events (e.g., a sudden unexpected health crisis or deterioration that changes the need for certain health services); financial implications of their inpatient admission; proximity to their health centre and most importantly, factors contributing to increased vulnerability (31, 37). Table 1 summarizes how these interventions can be implemented at different stages of in-patient care to support improved outcomes of hospitalized PLHIV.

**Table 1:** Key programmatic actions for supporting transitions in care for hospitalized PLHIV.

Stage of care	Key programmatic actions
Admission to inpatient care	- Establish an HIV diagnosis/verify HIV status
	- CD_4_ testing to identify advanced HIV disease or WHO Staging in the absence of CD_4_ testing
	- Referral to higher care centre where required
Inpatient stay	- Screen and identify major causes of admission
	- Relevant lab and radiological investigations
	- Establish a working diagnosis
	- Start relevant treatment, high quality nursing care, physical assistance, nutrition and review
	- Rapid ART if no contraindications are present
Planning for discharge	- Pre-discharge goal setting
	- Transitional care planning
	- Identify peer support groups/healthcare providers for post-discharge care
	- Identify needs for individualized support
During discharge	- Medication review
Post-discharge period	- Home visits, telephone calls, and support from social workers or community healthcare workers
	- Identification of post-illness morbidity, functional status of the individual, pain assessment and management where appropriate
	- Linkage to rehabilitation services among survivors of critical care
	- Provision of individualized support following needs assessment
	- ART initiation may be offered outside health facility if needed
	- Adherence support interventions (counselling, reminders, peer and other support, education)

HIV, Human immunodeficiency virus; ART, antiretroviral therapy; TB, tuberculosis; CD_4_, Cluster of differentiation 4 glycoprotein.

## 4 Conclusion

There remain significant concerns about how PLHIV being discharged from care can be successfully brought back into regular HIV care services. Recent WHO recommendations provide guidance to help reduce avoidable readmissions and improve retention in care. However, interventions that reduce mortality are still needed and this is an important knowledge gap for future research. Notwithstanding the recent reductions in global health financing, it is critical that programmes incorporate approaches to successfully re-engage individuals in regular care following a hospitalization to avert mortality during this high-risk period and ultimately achieve life-long viral suppression.
